# Crosstalk of mitochondrial dysfunction and macrophage polarization in sepsis

**DOI:** 10.3389/fimmu.2026.1692597

**Published:** 2026-02-20

**Authors:** Fuxi Ji, Li Zhang, Lili Ning, Min Zhang, Jingxiao Zhang

**Affiliations:** Department of Emergency and Critical Care Medicine, The Second Hospital of Jilin University, Changchun, China

**Keywords:** crosstalk, macrophage polarization, metabolic adaptation, mitochondrial dysfunction, sepsis

## Abstract

Sepsis is a complex condition marked by significant dysregulation of immune and metabolic processes, leading to multi-organ failure. Macrophages, key mediators of immune activity, demonstrate functional flexibility by switching between pro- and anti-inflammatory phenotypes in response to inflammatory and metabolic signals in their local environment. During sepsis, pathogen-derived signals activate host defense responses that impair intercellular oxygen transport, increase oxygen consumption by immune cells within inflamed tissues, and promote a metabolic transition toward aerobic glycolysis. This metabolic transition supports immune defense mechanisms, and the metabolic by-products further regulate immune activation through feedback in key signaling cascades, promoting a transition toward tolerance during the resolution phase. Since mitochondria are central hubs for cellular energy homeostasis, they play a crucial role in this process. Mitochondrial dysfunction and metabolic changes are now recognized as major contributors to the progression of sepsis. The accumulation of mitochondria-derived metabolites can further modulate immune signaling pathways, actively influencing macrophage function. Therefore, this review emphasizes the crosstalk between macrophage polarization and mitochondrial changes, with a focus on new molecular insights and the potential of mitochondrial pathways as biomarkers or therapeutic targets. These concepts provide a foundation for advancing both experimental research and clinical applications, potentially guiding future interventions to better manage sepsis and its associated mortalities.

## 1 Introduction

Sepsis is a severe, life-threatening condition caused by systemic organ failure that results from an abnormal and uncoordinated immune response to infection (1). It affects approximately 50 million people worldwide each year and remains a major cause of mortality, presenting a significant global health challenge (2, 3). Effective immune defense and host survival in sepsis rely on two evolutionarily conserved strategies: resistance and tolerance (4). Resistance processes focus on eliminating invading microorganisms, whereas disease tolerance enhances host viability by preventing and repairing tissue injury (5) caused by pathogenic insults and excessive inflammatory responses, without directly reducing the microbial burden (6). As key regulators of immune responses during sepsis, macrophages show functional adaptability, transitioning between pro-inflammatory and anti-inflammatory phenotypes in response to inflammatory signals and metabolic conditions in their surrounding environment (7). In addition to their roles in pathogen clearance, macrophages contribute to the maintenance of local and systemic homeostasis. Metabolic remodeling in macrophages is crucially involved in establishing disease tolerance and in developing immunosuppressive states during the later phases of sepsis (8).

Macrophage polarization in sepsis is regulated by both systemic metabolic conditions and local immune signals. Even under persistent inflammatory stimulation, changes in mitochondrial function and energy metabolism gradually drive macrophages through distinct transitional states, ultimately shifting from inflammatory to tissue-healing phenotypes. During sepsis, macrophages detect signals from the peripheral immune system and components from pathogens, while internal metabolic changes supply the energy required for effective immune responses. Simultaneously, this metabolic shift provides feedback regulation that modulates the intensity and duration of the inflammation, guides macrophages toward a tissue-healing profile, and reduces the risk of excessive immune activation and collateral tissue damage. Therefore, mitochondria play an essential role in maintaining cellular energy homeostasis and orchestrating immune responses, acting as key regulators of metabolic processes and inflammatory signaling pathways (9). Mitochondrial dysfunction, a key feature of sepsis, disrupts the electron transport chain (ETC), reduces ATP production, and increases the accumulation of reactive oxygen species (ROS) (10). Moreover, mitochondrial damage triggers the release of damage-associated molecular patterns (DAMPs), such as mitochondrial DNA, lipids, and metabolic intermediates, which synergize with pathogen-associated molecular patterns (PAMPs) to enhance immune activation (11).

Considering the crucial roles of metabolic remodeling and mitochondrial dysfunction in sepsis pathology, this review provides a detailed analysis of the bidirectional relationship between mitochondrial regulation and macrophage phenotype changes. Relevant literature was systematically retrieved from databases including PubMed, Web of Science, Scopus, Google Scholar, and the Cochrane Library, and publications were selected based on specific eligibility criteria.

## 2 Macrophage phenotypes and metabolic characteristics in sepsis

### 2.1 Influence of the inflammatory microenvironment on macrophage phenotypes in sepsis

Macrophages derive from two main sources: (1) fetal erythro-myeloid progenitors from the yolk sac, which persist into adulthood through self-renewal, and (2) bone marrow-derived monocytes from hematopoietic stem cells (12). Their phenotypic polarization is shaped by external signals and the local microenvironment, leading to distinct functional states (7, 13) (**Table 1**).

**Table 1:** The biological characteristics of macrophage subphenotype in inflammatory microenvironment.

Phenotype	Inducer	Cell markers		Secreted inflammatory mediators		Function
		Surface markers	Intracell markers	Cytokines	Chemokines	
M1	TNF-α, IFN-γ, IFN-β, LPS	CD36, CD68, CD80, CD86, IL-10↓, IL-12↑, MHC-II	COX2, iNOS, IRF5, STAT1	IL-6, IL-12, IL-17, IL-23, IL-27, TNF-α, IL-1β, ROS	CCL2-5, CXCL5, CXCL8-11, CXCL16, CCL15	Pro-inflammatory and defense against pathogens, Th1 response
M2a	IL-4, IL-10, IL-13	CD163, Dectin-1, MMR/CD206, IL-1RII	IRF4, STAT6, PPAR-γ	TGF-β, IL-10, IL-1Ra	CCL17, CCL18, CCL22, CCL26, CCL23	Anti-inflammatory, Tissue remodeling, Wound healing
M2b	IL-1β, TLR ligands, ICs	CD86, IL-12↓, IL-10↑	COX2, IRF4, SOCS3	IL-6, IL-10, TNF-α, IL-1β, GCSF, GM-CSF	CCL1, CCL20, CXCL1-3	Th2 activation, Immunoregulation
M2c	Glucocorticoids, TGF-β, IL-10	CD163, TLR-1, MMR/CD206	IRF4, SOCS3, TLR-8	IL-10, TGF-β	CCL16, CCL18, CXCL13	Immunoregulation, Tissue repair
M2d	Adenosine receptor ligands, TLR ligands	IL-10↑, IL-12↓, TNF-α↓		VEGF, IL-10, IL-12↓, TNF-α↓	CCL5, CXCL10, CXCL16	Angiogenesis, Clearance of apoptotic tissue

TNF-α, tumor necrosis factor α; IFN-γ, interferon γ; TGF-β, transforming growth factor β; LPS, lipopolysaccharides; MHC-II, major histocompatibility complex-II; COX, cyclooxygenase; iNOS, inducible nitric oxide synthase; IRF, interferon regulatory factors; STAT, signal transducers and activators of transcription; ROS, reactive oxygen species; Arg-2, arginase-2; PPAR-γ, peroxisome proliferator-activated receptor γ; FIZZI, resistin-like α. ICs, immune complexes; SOCS, suppressor of cytokine signaling; TLR-1, Toll-like receptor 1; VEGF, vascular endothelial growth factor; MMR, macrophage mannose receptor.

In sepsis, exposure to bacteria, viruses, fungi, or their fragments, as well as lipopolysaccharide (LPS), virulence determinants, and secreted bacterial proteins, and interferon-gamma (IFN-γ), promotes the polarization of macrophages into the M1 phenotype. These M1 macrophages display increased phagocytic activity and contribute to pathogen eradication and debris clearance by secreting pro-inflammatory factors like tumor necrosis factor-α (TNF-α), interleukins (IL-1, IL-6, IL-12), chemokines (CXCL9, CXCL10), and ROS, thus boosting the inflammatory response (14, 15). Excessive activation of M1 macrophages and the overproduction of pro-inflammatory mediators during the initial phase of sepsis can directly lead to multiple organ dysfunction syndrome and increased mortality, and they also contribute to immunosuppression and increased susceptibility to secondary infections during the later phase of sepsis (3, 12, 16). An endogenous “lactate clock” has been proposed in bacterially stimulated M1 macrophages, initiating specific gene expression programs to promote homeostasis (17). In the later stages of sepsis, anti-inflammatory cytokines such as IL-4, IL-10, IL-13, and transforming growth factor-β (TGF-β) mediate macrophage polarization toward the M2 phenotype, thus promoting immune regulation and tissue repair post-sepsis. In addition to the traditional M1/M2 classification, M2 macrophages can be further divided into subtypes (M2a, M2b, M2c, M2d) based on their specific functions, including resolving inflammation, promoting angiogenesis, facilitating tissue regeneration, and maintaining immune tolerance (7, 18). M2 macrophages help suppress immune responses by the secretion of IL-10 and TGF-β, enhancing tissue repair processes (19).

**Figure 1:**
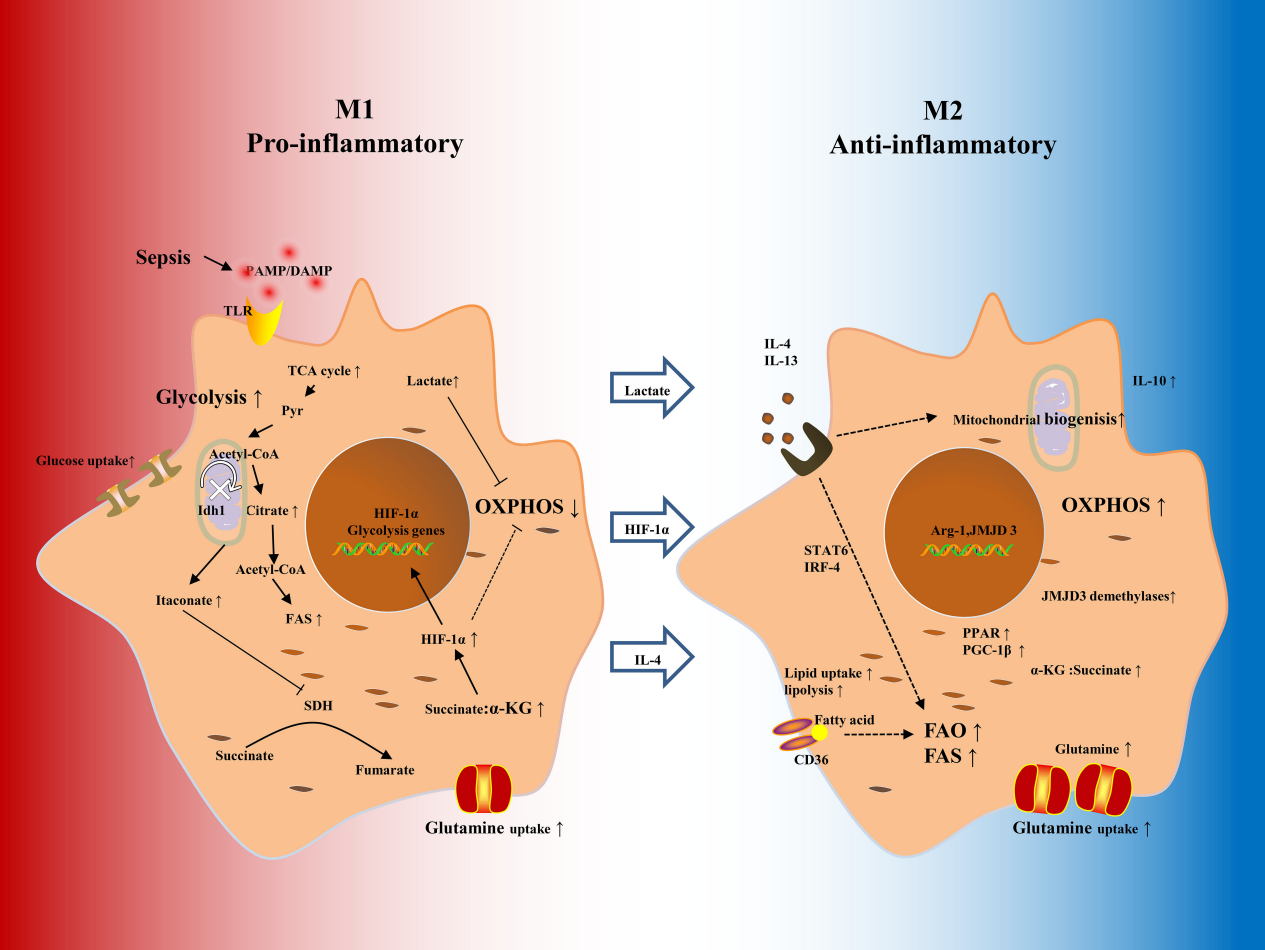
Metabolic reprogramming in macrophage polarization. Resting macrophages respond to PAMPs, DAMPs, and cytokines like IL-4 and IL-13 by differentiating into either classically activated M1 or activated M2 phenotypes. M1 cells shift their metabolism toward glycolysis, allowing rapid ATP generation and secretion of pro-inflammatory mediators. However, M2 cells show increased OXPHOS, FAO, and glutaminolysis, supporting anti-inflammatory actions and tissue repair. IDH catalyzes the conversion of isocitrate to α-KG; inhibiting this enzyme leads to citrate accumulation and the production of itaconate. Citrate-derived acetyl-CoA promotes histone acetylation, which facilitates inflammatory gene transcription and FAS. Itaconate suppresses SDH, blocking the conversion of succinate to fumarate, leading to succinate accumulation and affecting inflammatory signaling. A high succinate/α-KG ratio stabilizes HIF-1α, suppresses OXPHOS, and maintains glycolytic activity. M2 macrophages also increased FAO and glutamine consumption, with elevated CD36 level increasing uptake of triglyceride-rich lipoproteins. Lactate promotes M2 polarization *via* the HIF-1α axis and induces histone lactylation, contributing to immune equilibrium. IDH, isocitrate dehydrogenase; α-KG, α-Ketoglutaric acid; FAS, fatty acid synthesis; SDH, succinate dehydrogenase; HIF-1α, hypoxia inducible factor 1α; FAO, fatty acid oxidation; JMJD3, Jumonji domain-containing protein D3. Dashed lines indicate indirect causes, and solid lines indicate direct causes.

Macrophage metabolic states are closely related to their functional roles. Pro-inflammatory M1 macrophages predominantly rely on aerobic glycolysis to rapidly generate ATP, whereas anti-inflammatory M2 macrophages primarily depend on mitochondrial oxidative phosphorylation (OXPHOS) (**Figure 1**). During the early stage of sepsis, macrophages undergo a metabolic switch towards glycolysis to meet the increased energetic and biosynthetic needs of inflammation. Within 24 to 72 h after tissue injury, macrophages transition to an anti-inflammatory state, characterized by increased mitochondrial metabolic activity and decreased glycolytic gene expression, which supports tissue repair and restores homeostasis or induces immunosuppression (20). RNA viruses such as influenza, dengue, and norovirus are detected by sensors such as retinoic acid-inducible gene I (RIG-I), which triggers mitochondrial DNA (mtDNA) release and enhances type I interferon (IFN) production *via* the cGAS-STING pathway (21, 22). Similarly, DNA viruses such as herpesvirus stimulate mtDNA release to activate the innate antiviral response (23). Hypoxia promotes glycolysis and lactate production in macrophages (17). Hyperoxia also enhances M1 polarization, as evidenced by increased levels of inducible nitric oxide synthase (iNOS), IL-6, and IL-1β. Hyperoxia suppresses IL-4-induced M2 markers such as Arg1 and Fizz1, indicating that high oxygen levels may inhibit M2 macrophage polarization (24).

### 2.2 Metabolic reprogramming of macrophages in sepsis

In sepsis, macrophage activation is initiated by pathogen-derived factors and extracellular inflammatory signals, which induce the expression of genes associated with pro-inflammatory phenotypes. Intracellular metabolic reprogramming, primarily regulated by mitochondrial function, modulates both the strength and duration of the inflammatory response, directing the transition from pro-inflammatory to reparative macrophage states. This regulation is essential for preventing excessive inflammation and reducing collateral tissue damage. The most well-known metabolic profiles correspond to the M1 and M2 macrophage phenotypes, which are used here as representative models to illustrate the metabolic features of macrophages in sepsis.

#### 2.2.1 Metabolic characteristics of M1 macrophages

M1 macrophages primarily rely on glycolysis for their metabolism, supporting their inflammatory activity, and are characterized by overexpression of inducible nitric oxide synthase (iNOS) and elevated secretion of inflammatory mediators (25). Activation through LPS-Toll-like receptors (TLRs) interaction increases glucose uptake and enhances glycolytic flux, which is crucial for driving pro-inflammatory response (26). Hexokinase (HK), the rate-limiting enzyme of glycolysis, plays a pivotal role in M1 polarization. Pharmacological inhibition of HK substantially decreases M1 activation and reduces pro-inflammatory cytokine production (27). Mitochondrial function also contributes to M1 macrophage activation by affecting both metabolic pathways and inflammatory signaling. Silent information regulator 1 (SIRT1), a regulator of mitochondrial oxidative metabolism, has been shown to improve mitochondrial function and survival outcomes in mouse sepsis models (28). The pyruvate dehydrogenase (PDH) complex promotes mitochondrial glucose oxidation by converting pyruvate into acetyl-CoA, thus fueling the tricarboxylic acid (TCA) cycle (29). Inhibiting PDH kinase 1 (PDK1), which suppresses PDH activity, effectively prevents M1 polarization and reduces NOD-like receptor thermal protein domain-associated protein 3(NLRP3) inflammasome activation. This approach also preserves mitochondrial metabolism and increases survival in septic animals (30). The increased glycolysis in M1 macrophages further promotes flux through the pentose phosphate pathway (PPP), producing nicotinamide adenine dinucleotide phosphate oxidase (NADPH) needed for ROS production and supplying metabolic intermediates for biosynthesis (25).

**Figure 2:**
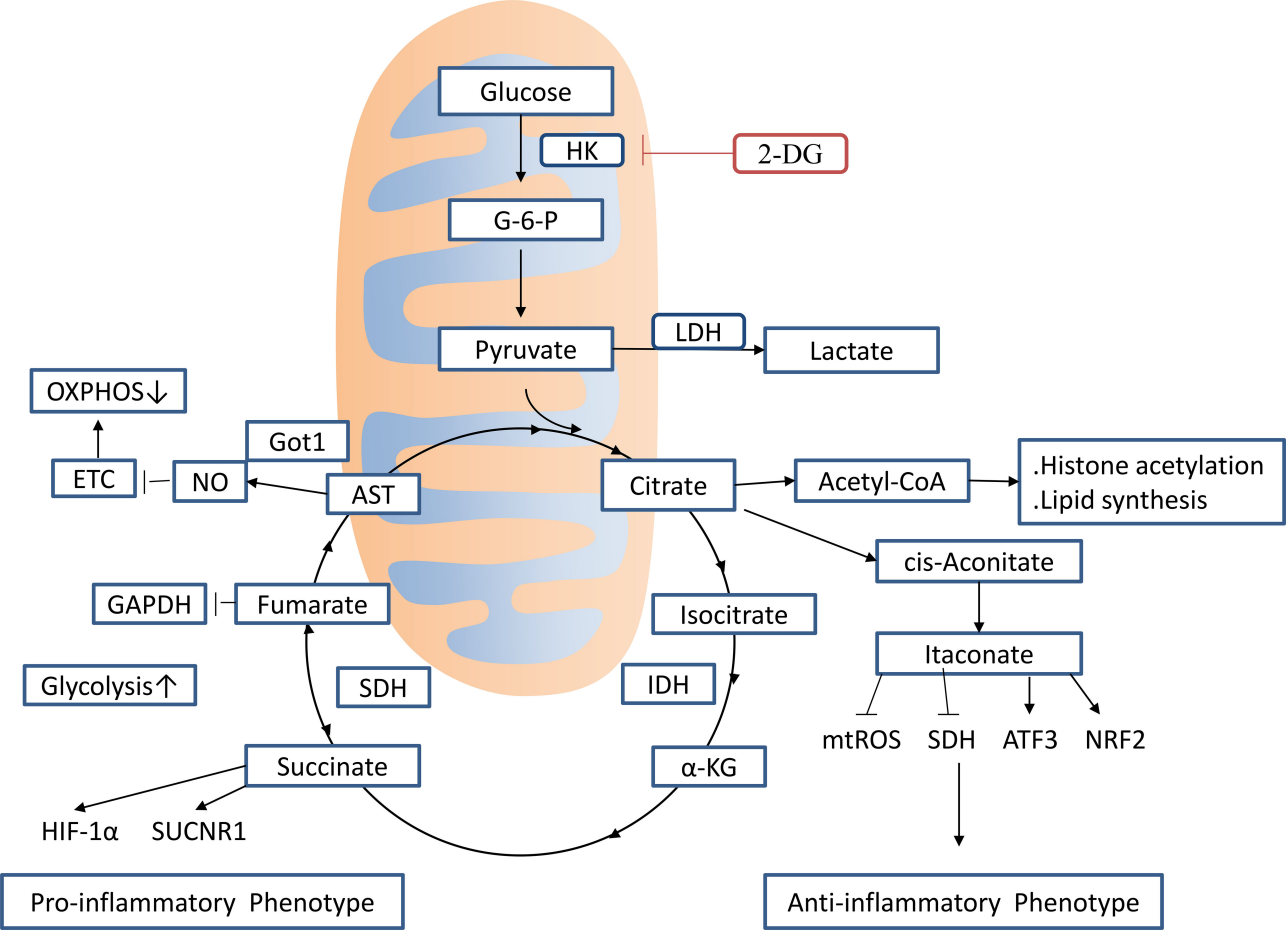
Effect of TCA intermediates on macrophage activation. Proinflammatory macrophages exhibit two breaks in the TCA cycle (at IDH and SDH), leading to the accumulation of citrate and succinate, and induction of the arginine-succinate shunt (AST) to support NO production. Itaconate, produced by the enzyme IRG1, exerts anti-inflammatory effects by inhibiting the activity of SDH and stimulating NRF2 and ATF3 induction. Fumarate, another TCA metabolite, is highly antimicrobial toward *L. monocytogenes* under acidic conditions by inhibiting the GAD system, which results in an intracellular pH increase. It also has an inhibitory effect on aerobic glycolysis by suppressing GAPDH activity. IRG1, immune-responsive gene 1; ATF3, activating transcription factor 3; IDH, isocitrate dehydrogenase; SDH, succinate dehydrogenase; HK, Hexokinase; 2-DG, 2-Deoxyglucose; GAD, glutamic acid decarboxylase; LDH, lactate dehydrogenase; SUCNR1, Succinate receptor 1; α-KG, α-Ketoglutaric acid; AST, arginine-succinate shunt; NRF2, Nuclear Factor erythroid 2-Related Factor 2.

Enhanced glycolytic activity elevates lactate production and diverts glucose-derived pyruvate into the TCA cycle, supporting energy metabolism and anabolic processes (31). This metabolic remodeling induces two main changes within the TCA cycle (**Figure 2**). First, citrate accumulates due to downregulation of isocitrate dehydrogenase (IDH), which is further used for itaconate synthesis (32). Citrate-derived acetyl-CoA promotes histone acetylation, activates inflammatory gene expression, and supports fatty acid synthesis (FAS), a process crucial for membrane biogenesis and protein synthesis (25, 31). Succinate Accumulation: Itaconate suppresses succinate dehydrogenase (SDH), blocking the conversion of succinate to fumarate and resulting in succinate accumulation. This accumulation modulates both metabolic and immune pathways (33). Increased succinate levels stabilize hypoxia-inducible factor 1-alpha (HIF-1α), maintaining inflammatory signaling and further driving metabolic reprogramming (25). This stage is characterized by decreased mitochondrial respiration and increased dependence on glycolysis, consistent with the pro-inflammatory phenotype of M1 macrophages (25). Simultaneously, itaconate has an anti-inflammatory function by lowering ROS and stabilizing transcription factors, activating transcription factor 3(ATF3) and nuclear factor erythroid 2-related factor 2(NRF2), thus reducing excessive inflammation (34). Succinate supports antimicrobial defense by inducing ROS generation. However, it also causes oxidative DNA damage and reduces intracellular NAD+ concentrations, forcing M1 macrophages to rely on salvage pathways to maintain NAD+ homeostasis and cellular viability (35). Moreover, nitric oxide (NO), produced by M1 macrophages, plays a bifunctional role. It shows antimicrobial activity but also inhibits M2 macrophage polarization, potentially delaying the resolution of inflammation (36). Moreover, NO disrupts cellular energy homeostasis by altering the ATP : ADP ratio, inhibiting ETC function, and reducing mitochondrial respiratory capacity, increasing the metabolic burden (32, 37).

#### 2.2.2 Metabolic characteristics of M2 macrophages

M2 macrophages are defined by increased mitochondrial OXPHOS, secretion of anti-inflammatory cytokines, and expression of arginase-1, all of which are essential for resolving inflammation and supporting Th2-mediated immune responses (38). Elevated arginase activity promotes the biosynthesis of ornithine and polyamines, among which spermidine plays a crucial role in regulating mitochondrial protein expression and initiating M2 macrophage differentiation (39).

##### 2.2.2.1 Fatty acid metabolism and glutamine dependency

Unlike M1 cells, M2 macrophages mainly depended on fatty acid oxidation (FAO) and glutamine metabolism rather than glycolysis to meet their metabolic requirements and maintain their anti-inflammatory functions (**Figure 1**) (40, 41). They show increased expression of genes related to lipid uptake, intracellular transport, and oxidative metabolism. IL-4 Stimulation increases lipid and glucose intake, indicating a metabolic adaptation that supports M2 activity (41). IL-4 also stimulates carbohydrate kinase-like protein (CARKL), an important regulator of PPP, promoting M2 polarization while reducing M1-rassociated responses (42). Glutamine metabolism is vital for M2 macrophages, supplying up to one-third of the carbon input into the TCA cycle, compared to about one-fifth in M1 macrophages (43, 44). Glutamine catabolism increases intracellular α-ketoglutarate (α-KG) levels, activating the histone demethylase jumonji domain-containing protein-3(JMJD3) and promoting transcription of M2-associated genes (44). IL-4 signaling further enhances histone acetylation at M2 gene loci *via* the Akt pathway. The involvement of mTOR complex 1 or 2 (mTORC1/2) varies by cellular context, which affects the M2 polarization (45). Furthermore, glutamine supports N-glycosylation of key surface receptors, including lectins and mannose receptors, which are crucial for effective immune surveillance and modulation (43).

##### 2.2.2.2 Lipid metabolism and mitochondrial function

M2 macrophages display enhanced expression of CD36, a scavenger receptor that mediates uptake of triglyceride-rich lipoproteins. This process is regulated by transcription factors such as STAT6, peroxisome proliferator-activated receptor-γ (PPAR-γ), PPAR-δ, PPAR-γ coactivator 1β (PGC-1β), and interferon regulatory factor 4 (IRF4) (41, 45). Lipid uptake, combined with fatty acid synthesis, enhances FAO and mitochondrial biogenesis, resulting in elevated oxygen consumption rates (OCRs) in M2 macrophages compared with M0 and M1 macrophages (41). However, studies using FAO inhibitors indicate that M2 polarization might not be strictly dependent on FAO, raising questions about whether these metabolic changes cause or result from the M2 phenotype (36, 46).

##### 2.2.2.3 Fatty acid synthesis

Fatty acid synthesis also plays a crucial role in shaping macrophage identity and function. Key transcriptional regulators, including sterol regulatory element-binding proteins (SREBPs) and liver X receptors (LXRs), influence macrophage phenotype specification by regulating lipid metabolic pathways. SREBPs promote lipid biosynthesis and favor M1 polarization, whereas LXRα supports M2 polarization through lipid metabolic regulation (47). Moreover, external lipid sources can induce PPAR-δ expression, which activates genes associated with apoptotic cell clearance and anti-inflammatory cytokine production (48).

## 3 Mitochondrial metabolic reprogramming and macrophage polarization in sepsis

In sepsis, persistent exposure to bacterial endotoxins and proinflammatory mediators impairs mitochondrial ATP production. It induces the release of DAMPs, including ROS, mitochondrial DNA (mtDNA), and cytochrome c (CYT-C), from damaged mitochondria. Similarly, intracellular accumulation of metabolic intermediates, such as itaconic acid and lactate, influences macrophage phenotypic transitions through various regulatory pathways. As the main sites of intracellular energy production, mitochondria act as a center for OXPHOS and inflammation regulation, thus playing a key role in macrophage phenotype regulation during sepsis. Excessive inflammatory signaling during the early phase of sepsis promotes rapid accumulation of metabolic byproducts (17, 49) (e.g., lactate, itaconate, etc), which drives a rapid transition of macrophages toward an immunosuppressive state. This transition accelerates systemic immunosuppression, raising the risk of secondary infections and poor clinical outcomes.

### 3.1 Macrophage polarization induced by the release of mitochondrial content

Macrophages apply various PRRs (**Table 1**), such as TLRs, CD36, CD68, CD80, CD86, MHC-II, and cytosolic NOD-like receptors, including NLRP3, to sense pathogenic or stress signals. During sepsis, the synergistic effect of microbial products, proinflammatory mediators, and tissue injury activates macrophages. A key factor in this process is mitochondrial dysfunction, which promotes the release of DAMPs, boosting immune responses and contributing to systemic organ dysfunction (11). As vital regulators of immunometabolism, mitochondria contain bacterial-like components that, when released into the extracellular space, act as potent activators of the innate immune system (50, 51). Mitochondrial byproducts, such as ROS, mtDNA, and cardiolipin, function as DAMPs, eliciting strong inflammatory responses. While these responses enhance host pathogen clearance, they can also exacerbate tissue damage and disease severity (51). Recent studies highlight mitochondrial impairment as a major driver of inflammation, functioning through key signaling pathways including TLR9, the cyclic GMP-AMP synthase (cGAS)-stimulator of interferon genes (STING) axis, and the NLRP3 inflammasome complex (51). mtROS and oxidized mtDNA (ox-mtDNA) serve as potent activators of the NLRP3 inflammasome, leading to caspase-1-mediated maturation of IL-1β and IL-18 and triggering pyroptotic cell death (52). These findings show the dual role of mitochondrial-associated signals in sepsis: while crucial for host antimicrobial defense, excessive activation can cause systemic inflammation.

### 3.2 Metabolic byproduct-mediated macrophage polarization in sepsis

During sepsis, disruption of TCA cycle integrity induces mitochondrial dysfunction, resulting in significant metabolic rewiring and the accumulation of key intermediates, including succinate (33), itaconate (53–55), α-KG (56, 57), and fumarate (58). These metabolites not only stimulate the NLRP3 inflammasome but also act as epigenetic regulators by altering histone modification patterns and controlling the expression of inflammation-related genes (56, 57) (**Figure 2**). As sepsis progresses, the release of mtDNA and other mitochondrial contents after mitochondrial permeability disruption is an important cause of persistent inflammatory damage (59).

#### 3.2.1 Pro-inflammatory mechanisms of mitochondrial-derived metabolites

##### 3.2.1.1 Succinate

During macrophage activation in sepsis, intracellular succinate accumulates and increases mitochondrial ROS production, serving as a signal to promote proinflammatory gene expression (25, 33). Oxidation of succinate through SDH stabilizes HIF-1α, partly via reverse electron transport (RET) and inhibition of PHDs. This activates glycolytic gene profiles and maintains the glycolytic metabolic phenotype of inflammatory macrophage (25). Moreover, extracellular succinate enhances inflammation by activating succinate receptor 1 (SUCNR1), leading to increased IL-1β production through an autocrine signaling loop (60). In intestinal tuft cells that express *SUCNR1*, succinate also triggers microbiota-driven type 2 immune responses against specific pathogens (61, 62).

##### 3.2.1.2 Citrate

As another intermediate of the TCA cycle, citrate is exported from mitochondria into the cytoplasm, where it acts as a precursor for acetyl-CoA synthesis, an essential substrate for lipid biosynthesis and histone acetylation (31, 63). This process is mediated by the mitochondrial citrate transporter and ATP citrate lyase, which converts citrate into acetyl-CoA and oxaloacetate. Acetyl-CoA promotes the transcriptional activation of proinflammatory genes and supports the synthesis of NO and prostaglandins (PG), particularly in response to TNF-α and IFN-γ. It also contributes to histone acetylation at IL-4-responsive loci in M2 macrophages and LPS-responsive loci in M1 macrophages (64). Oxaloacetate-generated NADPH is necessary for NO and ROS production (65). Furthermore, mitochondrial aconitase 2 converts citrate to cis-aconitate, which is further decarboxylated to produce itaconate (66). Itaconate inhibits inflammation by blocking SDH and reducing proinflammatory cytokine production (67). Citrate-derived metabolites play crucial roles in macrophage activation under LPS stimulation by regulating ROS, NO, PG, and itaconate synthesis, as well as histone acetylation and gene expression (68).

##### 3.2.1.3 NO

NO is a free radical produced from arginine through NO synthase (NOS). It can react with superoxide radicals to form reactive nitrogen species (RNS), which induce molecular and cellular damage (69). NO has various biological functions, including antimicrobial, immunomodulatory, cytotoxic, and antitumor effects (70). A dual role for NO has been identified: intracellular NO generated by iNOS can promote apoptosis in proinflammatory macrophages, whereas high concentrations of NO induced by LPS and IFN-γ exert anti-apoptotic effects in anti-inflammatory macrophages (71). This paradox may be due to the different metabolic effects of NO. In inflammatory macrophages, metabolic flux through the aspartate-argininosuccinate shunt supports arginine regeneration and NO synthesis. Glutamic-oxaloacetic transaminase 1 (GOT1), a key enzyme in this pathway, increases NO and IL-6 production while reducing mitochondrial respiration (43). NO also promotes mitochondrial-mediated apoptosis by releasing CYT-C into the cytosol (72). and directly regulates ETC activity by targeting subunits in Complex I (32) and Complex IV (73), leading to the inhibition of OXPHOS. These actions modify levels of TCA cycle intermediates, including citrate and succinate, and increase the production of the anti-inflammatory metabolite itaconate (32, 36), resulting in a decreased inflammatory response.

#### 3.2.2 Anti-inflammatory roles of mitochondria-derived metabolites

##### 3.2.2.1 Itaconate

Itaconate is generated within the mitochondrial matrix from cis-aconitate via the enzyme encoded by the immune-responsive gene 1 (IRG1) after LPS stimulation (49). It acts as a key regulator of macrophage function (67). Increasing evidence shows that itaconate suppresses immune activation by reducing proinflammatory gene expression and alleviating oxidative stress in activated macrophages (67, 74). Itaconate blocks the activity of SDH, a crucial enzyme in the TCA cycle, leading to succinate accumulation, decreased mitochondrial oxygen supply, and anti-inflammatory effects (67, 75). As an electrophile, itaconate covalently modifies cysteine residues on KEAP1, a cytosolic redox sensor that targets NRF2 for degradation (74). When KEAP1 is inhibited, Nrf2 becomes more stable and enters the nucleus, activating genes that reduce oxidative and inflammatory damage (76). Moreover, higher levels of itaconate following LPS stimulation facilitate NRF2 accumulation and activation of its antioxidant programs (74). Some studies have reported that the anti-inflammatory effects of itaconate can also be independent of NRF2, as its electrophilic nature can suppress IκBζ expression by inducing ATF3, thus specifically downregulating TLR-driven inflammatory gene expression (34, 77). A negative feedback loop between itaconate and IFN-I signaling has also been proposed, whereby interferon signaling increases IRG1 expression and itaconate production; the accumulated itaconate then suppresses IFN-I activity by reducing mitochondrial ROS and cytokine release, including IL-1β and IL-6 (67, 78).

##### 3.2.2.2 Fumarate

Fumarate, an intermediate in the TCA cycle, regulates macrophage activity. Under acidic conditions, it exhibits potent antimicrobial activity against E. coli and Listeria monocytogenes by inhibiting the GAD pathway, which converts glutamate into GABA and raises intracellular pH (79). Dimethyl fumarate (DMF), a synthetic fumarate derivative, is used clinically to treat inflammatory diseases like psoriasis and multiple sclerosis (80). DMF reduces aerobic glycolysis in activated myeloid and lymphoid cells by covalently modifying the catalytic cysteine residue of glyceraldehyde-3-phosphate dehydrogenase (GAPDH) (80). Both endogenous fumarate and exogenous DMF promote S-(2-succinyl)-cysteine formation on gasdermin D (GSDMD), modifying a key cysteine residue. Since GSDMD is involved in pyroptosis, succination hampers its interaction with caspase-1, decreasing GSDMD cleavage, oligomerization, membrane pore formation, and pyroptotic cell death (58). Recent research shows that NRF2 activators, such as 4-octyl itaconate (4-OI) and DMF, induce an interferon-independent antiviral response that broadly inhibits viral replication and reduces proinflammatory responses to various pathogenic viruses, including SARS-CoV-2 (81). These observations suggest that fumarate may share immunomodulatory effects with DMF, including suppression of aerobic glycolysis and potentially antimicrobial, antiviral, and anti-inflammatory activities.

##### 3.2.2.3 Lactate

Lactate serves as a key product of glycolysis, a major substrate for mitochondrial oxidative metabolism, and an intercellular signaling molecule that facilitates cell-cell metabolic communication (82). Many studies have shown that lactate affects macrophage metabolic reprogramming in various pathological conditions, including inflammation, cancer, and pulmonary fibrosis (83, 84). Lactate suppresses the M1 phenotype while promoting the anti-inflammatory, pro-angiogenic M2 phenotype through histone post-translational modifications (PTMs) and intracellular signaling pathways (85). Compared with other immunomodulatory metabolites such as itaconate, fumarate, and succinate, the sensing mechanisms and signaling pathways associated with lactate show advanced evolutionary adaptation (86). In sepsis, hypoxia and pathogen-induced anaerobic glycolysis lead to elevated lactate levels, which in turn further enhance lactylation processes (87). Lactate plays a central role in OXPHOS and glycolysis, functioning as a crucial metabolic intermediate. In 2014, Colegio et al. showed that tumor-associated macrophages (TAMs) in a melanoma model internalize lactate through monocarboxylate transporter 1 (MCT1), thus mediating polarization toward a tumor-promoting phenotype (88). Lactate promotes M2 polarization via HIF-1α activation and induces histone lactylation, further contributing to the maintenance of immune homeostasis (17, 89). These results highlight the multifaceted roles of mitochondrial metabolites in regulating inflammation and maintaining immune homeostasis, underscoring their potential as therapeutic targets for reducing sepsis-related damage.

## 4 Potential therapeutic approach: targeting mitochondria and macrophages in sepsis

### 4.1 Regulation of mitochondria and macrophages in the hyperinflammatory phase of sepsis

During the hyperinflammatory stage of sepsis, targeted inhibition of M1-like macrophage activation can significantly reduce the release of inflammatory mediators, alleviating tissue damage and decreasing mortality. Under physiological conditions, cellular energy metabolism primarily relies on OXPHOS and glycolysis. During sepsis, extensive metabolic alterations occur in nearly all cell types, involving a transition from OXPHOS to glycolytic metabolism, a process referred to as “metabolic reprogramming” (90). This transition is mainly caused by OXPHOS dysfunction, which compromises mitochondrial efficiency. In macrophages, although less efficient than OXPHOS, glycolysis supports rapid ATP production and provides metabolic intermediates required for immune activation and cell proliferation (91). Therapeutic strategies targeting glycolysis, including the use of 2-deoxyglucose (2-DG), have shown promise in reducing systemic inflammation in sepsis models, highlighting their potential clinical applications (92, 93). In sepsis models, the intervention with 2-DG and the fatty acid β-oxidation inhibitor esculetin suppressed M1 polarization and promoted M2 polarization of macrophages, both *in vivo* and *in vitro* (94) (**Table 2**).

**Table 2:** Therapeutic approaches targeting macrophage metabolic adaptations during sepsis.

Drugs	Category	Mode of action	Conclusion	Reference
DMM	succinate dehydrogenase inhibitor	DMM increases the expression of M2 macrophages-associated signature genes, including *Arg1*, *Ym1*, and Mrc1, decreases the expression of IL-1β, and enhances the levels of IL-10 in M1 macrophages through mitochondrial ROS-dependent STAT6 activation.	Promotes M2 Macrophage Polarization in inflammatory diseases	(95)
2-DG	glycolysis inhibitor	2-DG inhibits M1 polarization and promotes M2 polarization of macrophages *in vivo* and *in vitro*, inhibits the level of lactate and the expression of glycolysis-related genes (*Glut1*, *Hk2*, *Pfkfb1*, *Pkm*, and Ldha), and promotes the expression of fatty acid β-oxidation-related genes (*Cpt1a*, *Cpt2*, and *Acox1*).	2-DG rebalances M1/M2 macrophage polarization to treat sepsis-induced acute lung injury	(94)
Esculetin	fatty acid β-oxidation inhibitor	Esculetin inhibits M1 polarization and promotes M2 polarization of macrophages *in vivo* and *in vitro*, inhibits the level of lactate, and the expression of glycolysis-related genes (Glut1, Hk2, Pfkfb1, Pkm, and *Ldha*) and promotes the expression of fatty acid β-oxidation related genes (*Cpt1a*, *Cpt2*, and *Acox1*).	Esculetin rebalances M1/M2 macrophage polarization to treat sepsis-induced acute lung injury	(94)
4-OI	Nrf2 activators	4-OI decreases GAPDH activity, blocks glycolytic flux, downregulating aerobic glycolysis in activated macrophages in sepsis	4-OI reduces the mortality rate in the septic model and inhibits cytokine release	(96)
4-OI	NRF2 activators	4-OI activates the NRF2/HO-1 pathway, promoting macrophage polarization and attenuating inflammation in sepsis-induced myocardial dysfunction	4-OI promotes M2 macrophage polarization and attenuates inflammation in sepsis-induced myocardial dysfunction	(97)
4-OI	NRF2 activators	4-OI inhibits lipopolysaccharide-induced oxidative stress injury in macrophages and activates Nrf2 in the nucleus to hinder the expression of NF-κB p65, suppressing oxidative stress injury in sepsis-associated acute kidney injury.	4-OI can reduce the macrophage activation and oxidative stress injury in sepsis-associated acute kidney injury	(98)
DMF	NRF2 activators	DMF inhibits the phosphorylation of IκBα and IKK, as well as nuclear factor-κB (NF-κB) in macrophages upon LPS stimulation, consistent with the reduction in IL-10, IL-6, and TNF-α in serum.	DMF increases the survival of septic mice by 50% and attenuates organ damage	(99)
DMF	NRF2 activators	DMF significantly inhibits the cGAS-STING pathway to suppress microglia pyroptosis and reduce inflammatory cytokine levels in the hippocampus	DMF enhances survival rates and sepsis-associated encephalopathy	(100)

DMM, dimethyl malonate; 2-DG, 2-deoxyglucose; NRF2, Nuclear Factor erythroid 2-Related Factor 2; IL-1β, interleukin-1β; ROS, reactive oxygen species; STAT6, signal transducer and activator of transcription; GAPDH, glyceraldehyde-3-phosphate dehydrogenase; 4-OI, 4-octyl itaconate; NF-κB, nuclear factor-κB; TNF-α, Tumour necrosis factor-α; cGAS-STING, Cyclic GMP-AMP synthase; DMF, Dimethyl Fumarate.

Inhibition of mitochondrial succinate dehydrogenase (SDH) using Dimethyl Malonate promotes M2 macrophage polarization by enhancing STAT6 activation (95). KLF14, a novel Krüppel-like transcription factor, reduces glycolysis and inflammatory cytokine secretion in macrophages by inhibiting HK2 transcription of HK2 against sepsis in mice (101). The itaconate derivative, 4-octyl itaconate (4-OI), decreases aerobic glycolysis in activated macrophages by activating the NRF2/HO-1 pathway (97, 98), inhibits GAPDH activity (96), promotes M2 macrophage polarization, and reduces inflammation in sepsis-induced myocardial dysfunction and acute kidney injury (98). Dimethyl Fumarate (DMF), a new inhibitor of nitric oxide synthesis, increases septic mice survival by 50% and reduces organ damage through inhibition of IκBα and IKK phosphorylation, as well as NF-κB activity (99). DMF also prevents microglia pyroptosis in sepsis-associated encephalopathy by blocking the cGAS-STING pathway (100). Both DMF and 4-OI inhibit tissue factor-mediated coagulopathy by blocking the macrophage type I IFN-TF axis in LPS-induced sepsis and SARS-CoV-2 infection models (102).

### 4.2 Regulation of macrophages and mitochondria in the immune tolerance phase of sepsis

After excessive macrophage activation in the early hyperinflammatory stage of sepsis, the immune system progressively transitions into an immunosuppressive state. This immune tolerance increases the risk of recurrent and persistent hospital-acquired secondary infections, which can raise mortality rates (16, 103).

Therapeutic strategies to reverse sepsis-induced immun-osuppression remain exploratory. Several studies have shown that, in sepsis patients with immune dysregulation and secondary infections, agents such as β-glucan or monophosphoryl lipid A can reprogram macrophage metabolism by enhancing glycolysis, promoting M1 polarization, and facilitating pathogen clearance (104). Besides this, recombinant IL-7 has been reported to reverse sepsis-associated lymphopenia by activating the mTOR signaling pathway (105). Inhaled interferon-γ therapy has been shown to restore HLA-DR expression on alveolar macrophages and reduce the incidence of ventilator-associated pneumonia (106, 107). Similarly, early clinical trials of granulocyte-macrophage colony-stimulating factor (GM-CSF) in sepsis have shown improved HLA-DR expression on monocytes (108, 109). Despite these promising findings, the precise mechanisms of action, optimal therapeutic timing, and safety profiles of immune-activating interventions require further validation in large-scale randomized controlled trials.

## 5 Conclusion

This review highlights the close association between mitochondrial dysfunction and macrophage polarization in the pathogenesis of sepsis. Macrophage phenotypes are shaped by the combined influence of the extracellular inflammatory microenvironment and intracellular mitochondrial metabolic reprogramming. Although these metabolic adaptations are essential for mounting effective immune defenses, the accumulation of metabolic intermediates exerts feedback regulation on immune signaling pathways, promoting the transition toward anti-inflammatory states during the resolution phase. This dual role not only reduces tissue injury caused by excessive inflammation but also contributes to immunosuppression in the later stages of sepsis. Metabolic regulation is crucial for macrophage phenotypes, modulating mitochondrial energy metabolism. Current research primarily aims to attenuate excessive macrophage activation during the early hyperinflammatory phase of sepsis and to restore impaired phagocytic and antibacterial functions during the immunosuppressive phase. This review also highlights the dynamic interplay between macrophage polarization and mitochondrial alterations, elucidates emerging molecular mechanisms, and identifies mitochondrial pathways as potential therapeutic targets. Most studies focus on suppressing early-stage inflammatory responses and remain confined to animal models, whereas studies targeting macrophage dysfunction during late-stage sepsis are comparatively limited. The intricate interaction between extracellular inflammatory cues and intracellular mitochondrial metabolic remodeling represents a key regulatory mechanism underlying sepsis pathophysiology. Therapeutic strategies that modulate inflammation and cellular metabolism hold promise for improving sepsis outcomes; however, further mechanistic and clinical studies are required to establish their translational potential.
